# Surgically induced necrotizing scleritis after retinal detachment surgery masquerading as scleral abscess

**DOI:** 10.3205/oc000107

**Published:** 2019-05-29

**Authors:** V. G. Madanagopalan, Narayana Shivananda, Thiruvengadakrishnan Krishnan

**Affiliations:** 1 Vitreo-Retinal Services, Aravind Eye Hospital, Pondicherry, India; 2 Cornea Services, Aravind Eye Hospital, Pondicherry, India

**Keywords:** surgically induced necrotizing scleritis, vitrectomy, sclerotomy, retinal detachment surgery

## Abstract

Scleral necrosis is a rare occurrence after many ocular procedures. In the absence of infection or use of surgical adjuncts such as antimetabolites or radiation, the necrosis is presumed to be directly related to surgical trauma and is hence termed surgically induced necrotizing scleritis (SINS). A high index of suspicion is required for an early diagnosis of SINS and its differentiation from infective scleritis is important as the treatment modalities of these two related conditions are different. We report a case of SINS at sclerotomy site following 23-gauge transconjunctival retinal detachment surgery that was initially suspected to be a scleral abscess. Prompt recognition and institution of topical and systemic steroid therapy helped in limiting the extent of scleral damage.

## Introduction

Surgically induced necrotizing scleritis (SINS) is a delayed hypersensitivity reaction occurring after any ocular surgery involving scleral incisions. It has commonly been reported after pterygium excision and cataract surgery [1], [2]. Identification of SINS is essential as early institution of intensive steroid therapy is needed. In non-responsive cases, immunosuppressives may also be instituted. Besides, any future ocular surgery in these patients may predispose to recurrent necrotizing inflammation at the wound sites.

## Case description

A 27-year-old woman presented with defective vision in the left eye (OS) for two weeks. She had sustained an open globe injury in her right eye (OD) ten years ago and globe repair was performed. Further, cataract extraction was done in the left eye two years ago. She had no known systemic illnesses. Visual acuity was doubtful light perception in OD and 3/60 in OS. Clinical exam showed a pthysical right eye. Anterior segment exam of OS revealed aphakia and greyish pupillary reflex. Indirect ophthalmoscopy demonstrated a total rhegmatogenous retinal detachment (RD) in OS. Intraocular pressure (IOP) in OS was 10 mm Hg. 

A routine pre-surgical hematologic evaluation including total and differential blood counts, plasma glucose analysis, screening for Hepatitis B, HIV and syphilis was performed and no abnormalities were detected. Thereafter, she underwent 23-gauge transconjunctival RD surgery in a routine manner. A scleral buckle was not placed. At the conclusion of surgery, 360 degree endolaser was done. Silicone oil endotamponade was provided with 1000 cSt oil (Aurosil; Aurolabs, Madurai) and the sclerotomies were closed with 7-0 polyglactin sutures (Vicryl; Ethicon, New Jersey) placed in a shoelace pattern. 

**Figure 1:**
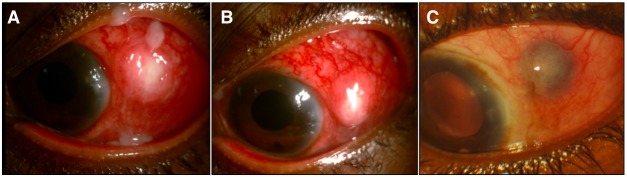
Surgically induced necrotizing scleritis at the sclerotomy site after vitrectomy. With intense conjunctival congestion, exudation at the sclerotomy site and presence of discharge on the fifth postoperative day, scleral abscess was suspected (A). After intense systemic and topical steroid therapy, resolving necrotizing scleritis with reduction in prominence of scleral induration can be seen at 1 week (B). At two months, scleritis has resolved completely with consequent scleral thinning (C). Uveal show can also be seen.

Five days after surgery, she presented with pain in the left eye, visual acuity in OS was 6/60 and IOP was 22 mm Hg. On examination, localized episcleral congestion and discharge were noted (Figure 1A (Fig. 1)). Discharge noted over the site of sclerotomy and at the lid margins was white in colour and was not frankly suppurative. Nevertheless, a high index of suspicion for infection was maintained since this was the early postoperative period. A scleral abscess was suspected and samples were sent for microbial analysis. Empirical antibiotic coverage with moxifloxacin 0.5% eye drops (Vigamox; Alcon, TX) six times a day and tobramycin 0.3% eye drops (Toba; Sun Pharma, Mumbai) six times a day was initiated. Topical prednisolone acetate 1% (Predforte; Allergan, Ireland) was used hourly. Timolol 0.5% eye drops (Iotim; FDC Ltd, Aurangabad) were used two times a day. 

Microbial analysis showed no organisms on microscopy or culture on agar plates (blood, chocolate, and Sabroud’s) and infusion broths. Hematologic investigations (complete blood count, plasma glucose and renal function tests) were unremarkable. In light of these laboratory reports, SINS was considered as the patient had undergone multiple ocular surgeries. Topical antibiotics were stopped. Topical prednisolone drops were tapered off in a weekly manner (8 times a day, 6 times a day, 4 times a day, 3 times a day and 2 times a day). A maintenance dose of topical prednisolone 2 times a day was maintained for a month. Oral prednisolone (Wysolone; Pfizer, Mumbai, India) was started at the dose of 40 mg once daily for a week. The dose was reduced in consecutive weeks to 30 mg, 20 mg, 10 mg and 5 mg in a tapering fashion. A maintenance dose of 5 mg was advised for a month.

With this regimen, the patient was relieved of symptoms in a week. Visual acuity was 6/60 and IOP was 20 mm Hg. Appreciable reduction in episcleral congestion was clinically made out. Although scleral necrosis was present, the nodule had decreased in size and the indurated margins were less prominent (Figure 1B (Fig. 1)). Over four weeks, the signs reduced remarkably and inflammation subsided considerably. At first month, visual acuity had improved to 6/24 and IOP was 18 mm Hg. After two months, visual acuity in the silicone oil filled aphakic eye was stable at 6/24. Scleral thinning was noted with uveal show (Figure 1C (Fig. 1)). IOP was maintained at 16 mm Hg with use of timolol eye drops.

## Discussion

Surgically induced necrotizing scleritis is a delayed hypersensitivity reaction due to sensitization to scleral antigens after ocular surgeries [1], [2]. Scleral necrosis may be precipitated by the use of antimetabolites or radiation during or immediately after surgery. Doshi et al. have noted that, of all cases in literature described as scleral necrosis, the most commonly performed primary surgeries were pterygium excision (63.4%) and cataract surgery (17.5%). However, when cases described particularly as SINS, where the necrosis was attributed to the primary surgery rather than the use of antimetabolites, radiation or infection was looked for, the authors reported that percentage was highest following strabismus surgery (90.9%) and vitrectomy (75%) [3]. Patients with SINS present with pain and redness at the surgical site. The time of onset varies between few days to many years [1], [4]. Extensive scleral necrosis may also present with a hypopyon. This is secondary to sterile intraocular inflammation or due to contiguous intraocular spread of infection [3].

In patients who develop SINS, organ specific auto-reactivity leads to capillary closure and tissue ischemia. Besides, surgery induced degradation of sclera by enzymes and damage to the epithelium could also contribute to SINS. On microscopy, the scleral tissue shows granulomatous inflammation and fibrinoid necrosis [5]. Contiguous intraocular structures such as the uveal tissue and retina may also be involved by the obliterative vasculitis. SINS is a clinical diagnosis after ruling out infective etiologies with microscopy and culture of wound swabs [1], [2], [3], [6]. Investigations for autoimmune diseases are necessary when scleral incisions have been used for cataract surgery. However, extensive investigations are less appropriate when SINS occurs in the setting of other ocular surgeries [3]. In the absence of inflammation, the sclera appears chalky white. When inflammation is prominent, differentiation from infective scleritis is difficult. With resolution of inflammation, a violaceous hue is imparted to the hitherto inflamed sclera since the underlying uveal tissue shows itself through the thinned out sclera. Few authors describe that SINS occurred after infectious scleritis [7], [8]. They hypothesize that release of inflammatory mediators by the infective agent may contribute to necrotizing scleritis [9]. On the other hand, what initially presents as SINS may get infected at a later stage [4], [10]. With compromised ocular coat integrity, this infection could spread to intraocular structures and cause devastating panophthalmitis [3].

Following vitrectomy, SINS can occur in any of the three sclerotomy ports used [2], [6]. Surgery for recurrent retinal detachment, use of cryotherapy, excessive diathermy, and placement of polyglactin sutures may be associated with sclerotomy site SINS following vitreoretinal procedures. When compared to anterior procedures, SINS following vitreoretinal surgery is rare and might require immunosuppression [2].

## Conclusion

When SINS is suspected, the importance of ruling out acute postoperative scleral infection is twofold. Firstly, on identification of aseptic necrosis rather than infection, the introduction of systemic and topical steroids will limit the spread of scleral necrosis. If necessary, antimetabolites may also be introduced. Secondly, care should be exercised as, in future surgeries, these patients are predisposed to necrosis of wound sites due to delayed hypersensitivity reaction.

## Notes

### Competing interests

The authors declare that they have no competing interests.
